# Silencing of OIP5-AS1 Protects Endothelial Cells From ox-LDL-Triggered Injury by Regulating KLF5 Expression via Sponging miR-135a-5p

**DOI:** 10.3389/fcvm.2021.596506

**Published:** 2021-03-12

**Authors:** Minghu Zhao, Yuanyuan Yang, Jingchao Li, Min Lu, Yu Wu

**Affiliations:** Department of Cardiovascular Comprehensive Ward II, Henan Provincial People's Hospital, Zhengzhou, China

**Keywords:** AS, OIP5-AS1, miR-135a-5p, KLF5, ox-LDL, cytotoxicity

## Abstract

**Background:** Long non-coding RNAs (lncRNAs) have been implicated in the pathogenesis of atherosclerosis. LncRNA OIP5 antisense RNA 1 (OIP5-AS1) has been found to be associated with the development of atherosclerosis. In this study, we further investigated the molecular basis of OIP5-AS1 in atherosclerosis pathogenesis.

**Methods:** Oxidative low-density lipoprotein (ox-LDL) was used to treat human umbilical vein endothelial cells (HUVECs). The levels of OIP5-AS1, miR-135a-5p, and Krüppel-like factor 5 (KLF5) were detected by quantitative real-time polymerase chain reaction (qRT-PCR) or western blot. Cell viability, migration, and apoptosis were evaluated using the Cell Counting Kit-8 (CCK-8), Transwell, and flow cytometry, respectively. The levels of interleukin-6 (IL-6), tumor necrosis factor-α (TNF-α), and malondialdehyde (MDA) were determined with enzyme-linked immunosorbent assay (ELISA). Targeted interactions among OIP5-AS1, miR-135a-5p, and KLF5 were confirmed by dual-luciferase reporter and RNA immunoprecipitation (RIP) assays. Animal studies were performed to assess the role of OIP5-AS1 in atherosclerosis progression *in vivo*.

**Results:** Our data showed the significant upregulation of OIP5-AS1 in atherosclerosis serum and ox-LDL-stimulated HUVECs. The silencing of OIP5-AS1 protected against ox-LDL-triggered cytotoxicity in HUVECs and diminished lipids secretion in ApoE^−/−^ mice. Moreover, OIP5-AS1 functioned as a molecular sponge of miR-135a-5p, and miR-135a-5p was a functional mediator of OIP5-AS1 in regulating ox-LDL-induced HUVEC injury. KLF5 was a direct target of miR-135a-5p, and the increased expression of miR-135a-5p alleviated ox-LDL-induced cytotoxicity by downregulating KLF5. Furthermore, OIP5-AS1 influenced KLF5 expression through sponging miR-135a-5p.

**Conclusion:** The current work identified that the silencing of OIP5-AS1 protected against ox-LDL-triggered cytotoxicity in HUVECs at least in part by influencing KLF5 expression via acting as a miR-135a-5p sponge.

## Introduction

Atherosclerosis, a chronic inflammatory disorder, is the pivotal pathological process of cardiovascular disease (CVD) (1, 2). Dysfunction of endothelial cells plays an important role in the progression of atherosclerosis (3). Oxidative low-density lipoprotein (ox-LDL) enhances the occurrence and development of atherosclerosis through various mechanisms, including the induction of the dysfunction of endothelial cells (4). A clearer understanding of how ox-LDL drives endothelial cell injury is very important for developing the effective approaches to inhibit atherosclerosis progression.

Long non-coding RNAs (lncRNAs) are >200-nucleotide RNAs that are implicated in diverse biological and pathological processes (5). Many lncRNAs have recently been shown to be involved in atherosclerosis pathogenesis by functioning as microRNA (miRNA) sponges (6). For instance, Wu et al. demonstrated that the deficiency of taurine upregulated gene 1 (TUG1) protected against ox-LDL-triggered cytotoxicity in human umbilical vein endothelial cells (HUVECs) by targeting miR-148b (7). Li et al. reported that metastasis-associated lung adenocarcinoma transcript 1 (MALAT1) acted as a miR-155 sponge to suppress atherosclerosis inflammatory response induced by ox-LDL (8). As for OIP5 antisense RNA 1 (OIP5-AS1), it was discovered to be associated with ox-LDL-mediated atherosclerosis progression in endothelial cells (9). Although the OIP5-AS1/miR-320a/lectin-like ox-LDL receptor 1 (LOX1) network has been highlighted in atherosclerosis pathogenesis (10), our understanding of the molecular basis of OIP5-AS1 remains limited.

MiRNAs are important regulators in atherosclerosis pathogenesis by silencing target genes (11, 12). MiR-135a-5p, an underexpressed miRNA in atherosclerosis, was reported to exert a potential anti-atherosclerotic activity in HUVECs under ox-LDL stimulation (13). When we used computational methods to help identify the molecular basis of OIP5-AS1, we found two putative binding sequences among OIP5-AS1, miR-135a-5p, and Krüppel-like factor 5 (KLF5). For these reasons, we undertook to investigate the involvement of the lncRNA/miRNA/mRNA regulatory network in ox-LDL-triggered HUVEC damage.

## Materials and Methods

### Patient Cohort and Serum Samples

In this project, 41 patients with atherosclerosis were enrolled from Henan Provincial People's Hospital, and 25 healthy individuals without any history of cardiovascular, inflammatory, or other diseases were recruited as normal controls. The clinicopathologic features of these subjects were provided in Table 1. Five milliliters of peripheral blood was collected from each participant, and serum samples were stored at −80°C. Ethics approval for the project was obtained from the Human Research Ethics Committee of Henan Provincial People's Hospital, and written informed consent was provided by all of the subjects.

**Table 1:** Clinicopathologic features of atherosclerosis patients and healthy controls.

**Parameters**	**Control group (*n* = 25)**	**Atherosclerosis group (*n* = 41)**
Gender (male/female)	11/14	19/22
Age (years)	51.6 ± 4.9	58.3 ± 6.9
LDL-C (mg/dl)	101.6 ± 20.5	139.5 ± 33.2^*^
HDL-C (mg/dl)	38.2 ± 6.8	32.5 ± 8.6^*^
T.CHOL (mg/dl)	148.9 ± 42.3	196.3 ± 52.6^*^
BMI	23.9 ± 1.2	28.3 ± 1.8

*BMI, body mass index; HDL-C, high-density lipoprotein cholesterol; LDL-C, low-density lipoprotein cholesterol; T.CHOL, total cholesterol*.

* *P < 0.05 by chi-square test*.

### Cell Culture and Treatment

HUVECs (ATCC® PCS-100-013™) and human aortic endothelial cells (HAECs, ATCC® PCS-100-011) were purchased from the American Type Culture Collection (ATCC, Manassas, VA, USA) and propagated in endothelial basal medium (EBM-2, Lonza, Basel, Switzerland) with 10% fetal bovine serum (EuroClone, Milan, Italy) at 37°C in a 5% CO_2_ humidified atmosphere. The sixth to seventh generations were used for our study. To generate the AS cell model *in vitro*, HUVECs of ~50% confluence were cultured in the complete medium containing various concentrations (20, 40, and 80 μg/ml) of ox-LDL (Yesen, Shanghai, China) for 24 h or 40 μg/ml of ox-LDL for 12, 24, and 48 h.

### Quantitative Real-Time Polymerase Chain Reaction (qRT-PCR)

Total RNA was extracted from serum samples (500 μl) and cultured cells using a modified TRIzol co-purification technique as previously reported (14). Briefly, for each 500 μl of serum and cell suspension, phase separation was done by adding 2 ml of TRIzol (Invitrogen, Hemel Hempstead, UK), followed by the addition of 200 μl of 1-bromo-4-methoxybenzene (Invitrogen). Total RNA was washed with 75% ethanol before solubilization with 50 μl of nuclease-free water. To quantify the expression of OIP5-AS1, KLF5, and endogenous control glyceraldehyde-3-phosphate dehydrogenase (GAPDH), 1 μg of RNA was reverse transcribed to cDNA using the PrimeScript™ RT Reagent Kit (TaKaRa, Beijing, China), and qRT-PCR was performed using the One Step TB Green® PrimeScript™ PCR Kit (TaKaRa) as recommended by the manufacturers. The quantification of miR-135a-5p and U6 reference gene was performed using the Mir-X miRNA cDNA Synthesis Kit for cDNA synthesis and Mir-X miRNA qRT-PCR TB Green® Kit for qRT-PCR as per the protocols of the manufacturer (TaKaRa). The amplification profile was denatured at 95°C for 10 min, followed by 40 cycles of 95°C for 20 s and 60°C for 1 min. All reactions were run in triplicate on a LightCycler 480 (Roche Diagnostic, Sussex, UK). The primers used in PCR amplification were provided in Table 2. Fold change of gene was calculated by the 2^−ΔΔCt^ method (15).

**Table 2:** Primer sequences of qRT-PCR.

**Primers for PCR (5′-3′)**		
OIP5-AS1	Forward	TGCGAAGATGGCGGAGTAAG
	Reverse	TCACAGGATGAGCCAGGATTT
miR-135a-5p	Forward	AACCCTGCTCGCAGTATTTGAG
	Reverse	GCGGCAGTATGGCTTTTTATTCC
KLF5	Forward	ACACCAGACCGCAGCTCCA
	Reverse	TCCATTGCTGCTGTCTGATTTGTAG
GAPDH	Forward	GCACCACCAACTGCTTAGCA
	Reverse	GTCTTCTGGGTGGCAGTGATG
U6	Forward	CTCGCTTCGGCAGCACA
	Reverse	AACGCTTCACGAATTTGCGT

### Subcellular Localization Assay

Total cytoplasmic RNA and nuclear RNA were isolated from HUVECs using the Cytoplasmic & Nuclear RNA Purification Kit (Norgen Biotek, Thorold, ON, Canada) as per the accompanying protocols. U6 and GAPDH were applied as the nuclear and cytoplasmic reference genes, respectively.

### Transient Transfection of Cells

For OIP5-AS1 silencing *in vitro* studies, HUVECs were transiently transfected with siRNA specific to OIP5-AS1 (si-OIP5-AS1, 30 nM; RiboBio, Guangzhou, China) or negative control siRNA (si-NC) using Lipofectamine 3000 (Invitrogen) based on the manufacturer's suggestion. For the overexpression studies, HUVECs were transfected with pcDNA-based recombinant-overexpressing plasmid specific to OIP5-AS1 or KLF5 (100 ng; RiboBio), with the non-target pcDNA plasmid as a negative control. MiR-135a-5p overexpression or knockdown cells were generated by using a miR-135a-5p mimic, the inhibitor of miR-135a-5p (anti-miR-135a-5p, 30 nM; RiboBio), or the corresponding scrambled oligonucleotide sequences (miR-NC mimic or anti-miR-NC) using Lipofectamine 3000. After 24 h of transfection, the cells were harvested for further analyses.

### Cell Viability and Apoptosis Assays

After various transfections, HUVECs were stimulated with 40 μg/ml of ox-LDL for 24 h. Cell viability assay was done by adding 10 μl of Cell Counting Kit-8 (CCK-8) reagent into each well and by incubating at 37°C for 4 h based on the recommendations of the manufacturers. Cell viability was proportional to the absorbance at 450 nm using a microplate reader (Thermo Fisher Scientific, Paisley, UK). Cell apoptosis assay was carried out by double staining with 5 μl of fluorescein isothiocyanate (FITC)-labeled Annexin-V and 2 μl of propidium iodide (PI) as per the directions of the manufacturers (KeyGen Biotech, Nanjing, China). The apoptotic cells were scored using a FACScan flow cytometer (BD Biosciences, Cowley, UK).

### Transwell Migration Assay

Twenty-four Transwell inserts (8-μm pore size, BD Biosciences) were used for cell migration assays. Briefly, 2.5 × 10^4^ treated HUVECs were plated in the top chamber, and a medium containing 10% serum was added in the lower chamber as a chemoattractant. The cells were stained with 0.5% crystal violet after 24 h of incubation and counted under a microscope at 100 × magnification.

### Enzyme-Linked Immunosorbent Assay (ELISA)

The cellular levels of interleukin-6 (IL-6) and tumor necrosis factor-α (TNF-α) were tested using human IL-6 and TNF-α ELISA kits, respectively, based on the suggestion of the manufacturer (Abcam, Cambridge, UK). Malondialdehyde (MDA) quantification in HUVECs was done with a Human MDA Assay Kit (Elabscience, Wuhan, China) as recommended by the manufacturers.

### Bioinformatics, Dual-Luciferase Reporter and RNA Immunoprecipitation (RIP) Assays

The miRNAs that potentially bound to OIP5-AS1 and the putative targets of miR-135a-5p were predicted using starBase v.2 software. The fragments of OIP5-AS1 harboring the wild-type and mutated miR-135a-5p-binding sites were synthesized by BGI (Shenzhen, China) and individually cloned into the pmirGLO vector (Promega, Mannheim, Germany) to produce OIP5-AS1 wild-type and mutant-type luciferase reporter (WT-OIP5-AS1 or MUT-OIP5-AS1). The segments of KLF5 3′UTR harboring target sequence or mismatched miR-135a-5p complementary sequence (BGI) were individually inserted into the pmirGLO vector to generate KLF5 3′UTR wild-type or mutant-type luciferase reporter (KLF5 3′UTR-WT or KLF5 3′UTR-MUT). HUVECs of 50% confluence were transiently transfected with each reporter construct (100 ng) and miR-135a-5p mimic or negative control mimic. Cell extracts were prepared with radioimmunoprecipitation assay (RIPA) lysis buffer (Elabscience) 36 h after transfection, and the ratio of *Renilla* to firefly luciferase was determined using the dual-luciferase assay system (Promega).

For RIPAs, HUVECs were homogenized in ice-cold RIPA lysis (Invitrogen). Cell lysates (10 μl) were then incubated with protein A/G bead-coupled anti-Argonaute2 (anti-Ago2, Abcam; dilution 1:50) or isotype anti-IgG (Abcam; dilution 1:100) antibody at 4°C for 6 h. The beads were harvested, and total RNA was isolated to measure OIP5-AS1, miR-135a-5p, and KLF5 enrichment levels by qRT-PCR.

### Western Blot

Western blot analysis was performed as described previously (16). Briefly, 50 μg per lane of cellular protein or 10 μl per lane of serum sample was resolved on a 12% SDS polyacrylamide gel and electro-transferred onto an Immobilon-P membrane (Millipore, Shanghai, China). The membranes were probed with primary antibodies against KLF5 (1:1,000, Abcam), Bax (1:1,000, Abcam), Bcl-2 (1:1,000, Abcam), and GAPDH (1:2,000, Abcam), followed by the incubation with IgG secondary antibody conjugated with horseradish peroxidase (1:3,000, Abcam). Protein bands were detected using enhanced chemiluminescence (Amersham Biosciences, Freiburg, Germany), and densitometry was quantified with the ImageJ software (16).

### Lentiviral Particle Generation

Lentiviral constructs harboring OIP5-AS1-shRNA (sh-circRERE) or scrambled shRNA (sh-NC) were obtained from Genomeditech (Shanghai, China). The lentiviral constructs were individually transfected into 293T cells (ATCC) with pMD2.G and psPAX2 (Addgene, Cambridge, MA, USA) to generate virus particles. Virus-containing suspension was purified with sterile filters (0.22 μm) and stored at −80°C for further experiments.

### Animal Studies

All animal procedures were performed according to the Academia Sinica IACUC and Council of Agriculture Guidebook for the Care and Use of Laboratory Animals, and the study was approved by the Ethics Committee of Henan Provincial People's Hospital. Male 8-week-old ApoE^−/−^ mice (*n* = 18) and wild-type C57BL/6J mice (*n* = 6) were purchased from the Vital River Laboratory (Beijing, China) and housed in a specific-pathogen-free environment in the animal facility of the Institute of Biomedical Sciences, Academia China. ApoE^−/−^ mice were fed with a high-fat diet (15.8% fat and 1.25% cholesterol), and wild-type C57BL/6J mice were fed with normal chow. In the 6th week, the ApoE^−/−^ mice were randomly divided into three groups: control, sh-NC group, or sh-OIP5-AS1 group (*n* = 6 per group). The lentiviral particles (50 μl) were injected into the ApoE^−/−^ mice by a caudal vein every week until the 12th week, and the control group was injected with the same volume of PBS. At the end of the experiments, blood samples were collected from these mice, and serum samples were harvested for further analyses. The expression levels of OIP5-AS1, miR-135a-5p, and KLF5 were gauged by qRT-PCR and western blot as above. The levels of total cholesterol, triglyceride, high-density lipoprotein cholesterol (HDL-C), and low-density lipoprotein cholesterol (LDL-C) were detected using commercially available enzyme kits per the manufacturer's instructions (Nanjing Jiancheng Bioengineering Institute, Nanjing, China).

### Statistical Analysis

Data were expressed as the mean ± standard deviation (SD) from at least three independent biological replicates. Student's *t*-test or analysis of variance (ANOVA) was used to compare differences of different independent groups of quantitative data. The correlation of miR-135a-5p expression and OIP5-AS1 or KLF5 level was measured by the Spearman rank correlation. The clinical parameters between atherosclerosis patients and healthy controls were compared by a chi-square test. All *P*-values were two-tailed, and those <0.05 were considered a statistically significant difference.

## Results

### OIP5-AS1 Was Overexpressed in Atherosclerosis Serum and Ox-LDL-Stimulated HUVECs

**Figure 1:**
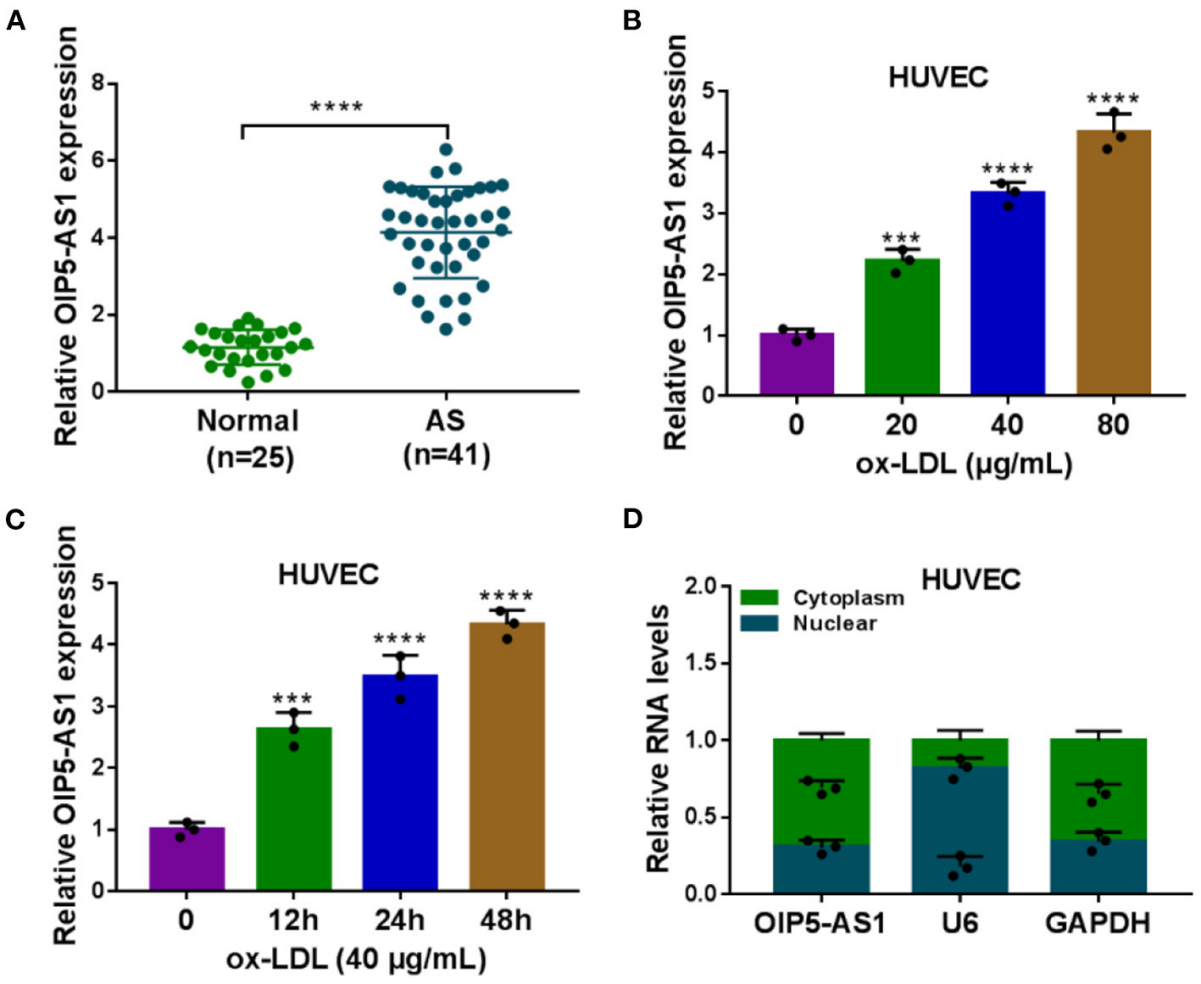
OIP5-AS1 was highly expressed in atherosclerosis serum and ox-LDL-stimulated HUVECs. **(A)** Relative OIP5-AS1 expression by qRT-PCR in 41 atherosclerosis serum samples and 25 normal controls. Error bars represented SD; ****P < 0.0001 by Student's *t*-test. **(B,C)** Relative OIP5-AS1 expression by qRT-PCR in HUVECs treated with 20, 40, and 80 μg/mL of ox-LDL and with 40 μg/ml of ox-LDL treatment for 12, 24, and 48 h. *n* = 3 independent biological replicates; error bars represented SD; ***P < 0.001, ****P < 0.0001 by one-way ANOVA with Tukey's *post hoc* test. **(D)** Subcellular localization assays in HUVECs. *n* = 3 independent biological replicates; error bars represented SD.

Firstly, we evaluated the expression of OIP5-AS1 in the serum samples of atherosclerosis patients and ox-LDL-stimulated HUVECs. As demonstrated by qRT-PCR, OIP5-AS1 level was upregulated in atherosclerosis serum samples compared with the normal controls (*P* < 0.0001, Figure 1A). Moreover, ox-LDL stimulation led to an upregulation in the expression of OIP5-AS1 in HUVECs in dose- and time-dependent manners (*P* < 0.001 or *P* < 0.0001, Figures 1B,C). Furthermore, subcellular localization assays showed that OIP5-AS1 was mainly present in the cytoplasm of HUVECs (Figure 1D).

### Silencing of OIP5-AS1 Alleviated Ox-LDL-Triggered Injury in HUVECs

**Figure 2:**
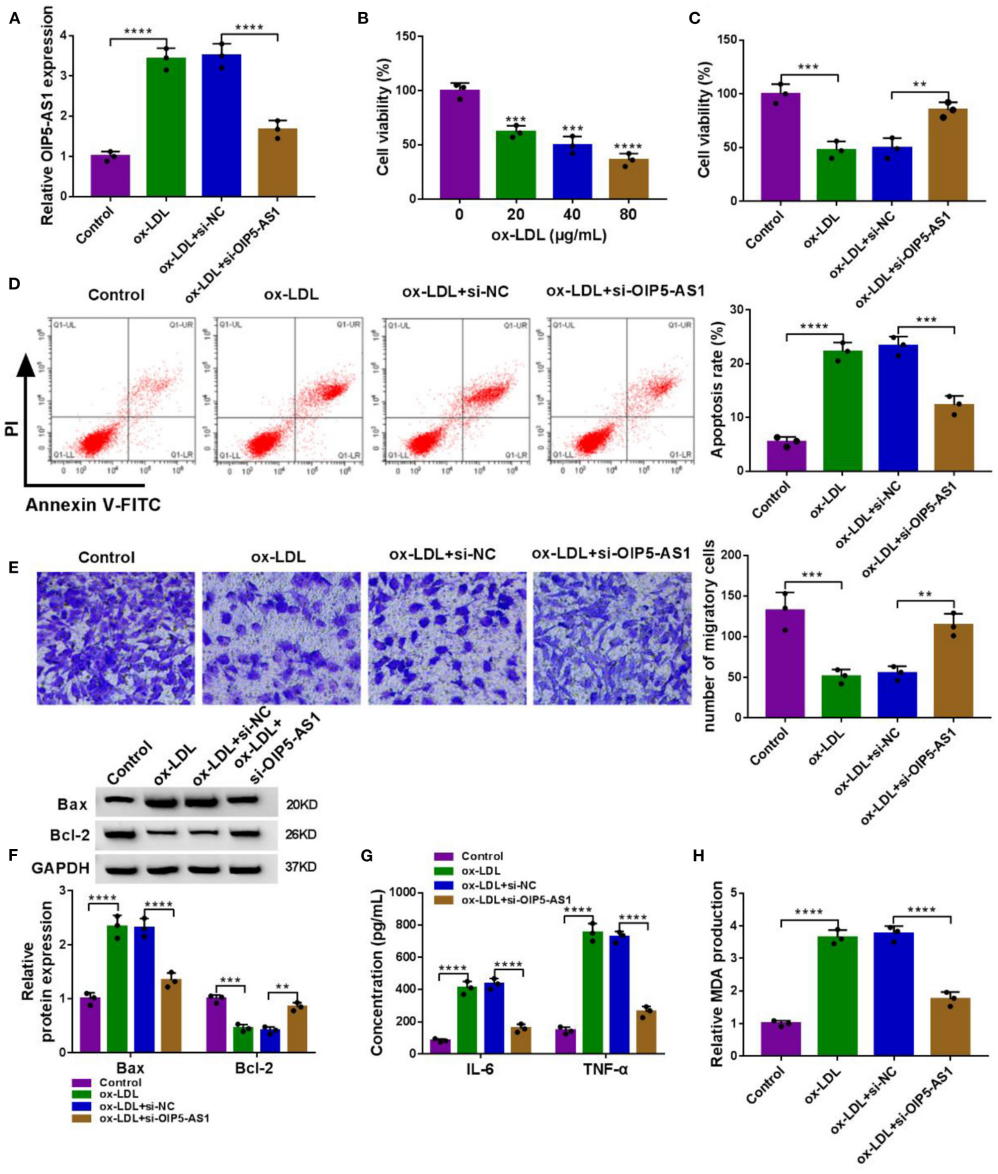
OIP5-AS1 silencing relieved ox-LDL-triggered cytotoxicity in HUVECs. **(A)** OIP5-AS1 expression by qRT-PCR in HUVECs transfected with or without si-OIP5-AS1 or si-NC before ox-LDL stimulation (40 μg/ml, 24 h). *n* = 3 independent biological replicates; error bars represented SD; ****P < 0.0001 by one-way ANOVA with Tukey's *post hoc* test. **(B)** CCK-8 assay for cell viability in HUVECs stimulated with 20, 40, and 80 μg/ml of ox-LDL for 24 h. *n* = 3 independent biological replicates; error bars represented SD; ***P < 0.001 or ****P < 0.0001 by one-way ANOVA with Tukey's *post hoc* test. CCK-8 assay for cell viability **(C)**, flow cytometry for cell apoptosis **(D)**, Transwell assay for cell migration **(E)**, western blot for Bax and Bcl-2 levels **(F)**, ELISA for IL-6, TNF-α, and MDA levels **(G,H)** in HUVECs transfected with or without si-OIP5-AS1 or si-NC before 40 μg/ml of ox-LDL stimulation for 24 h. *n* = 3 independent biological replicates; error bars represented SD; **P < 0.01, ***P < 0.001, or ****P < 0.0001 by one-way ANOVA with Tukey's *post hoc* test.

In addition to the increased effect on OIP5-AS1 expression (Figure 2A), ox-LDL inhibited cell viability (*P* < 0.01, *P* < 0.001, or *P* < 0.0001, Figures 2B,C), enhanced cell apoptosis (*P* < 0.0001, Figure 2D), and suppressed cell migration (*P* = 0.0006, Figure 2E). Moreover, ox-LDL elevated pro-apoptotic protein Bax expression (*P* < 0.0001) and reduced anti-apoptotic protein Bcl-2 level (*P* = 0.0009) (Figure 2F), supporting the enhancement of ox-LDL on cell apoptosis. Additionally, ox-LDL resulted in increased levels of pro-inflammatory cytokines IL-6 and TNF-α (*P* < 0.0001, Figure 2G) and oxidative stress marker MDA (*P* < 0.0001, Figure 2H) in HUVECs.

To determine the biological role of OIP5-AS1 in atherosclerosis pathogenesis, we performed loss-of-function analyses by silencing OIP5-AS1 with specific siRNA (si-OIP5-AS1). Transient transfection of si-OIP5-AS1, but not the si-NC control, led to a significant reduction in OIP5-AS1 level in ox-LDL-stimulated HUVECs (*P* < 0.0001, Figure 2A). Functional analyses revealed that the knockdown of OIP5-AS1 abolished ox-LDL-mediated anti-viability (*P* = 0.0037, Figure 2C), pro-apoptosis (*P* = 0.0001, Figure 2D), and anti-migration (*P* = 0.0057, Figure 2E) effects in HUVECs. Moreover, OIP5-AS1 silencing abrogated the impact of ox-LDL on Bax, Bcl-2, IL-6, TNF-α, and MDA expression (*P* < 0.01 or *P* < 0.0001, Figures 2F–H) in HUVECs. As the control, the enforced expression of OIP5-AS1 in HUVECs using its overexpressing plasmid inhibited cell viability, enhanced cell apoptosis, and promoted IL-6, TNF-α, and MDA expressions (Supplementary Figures 2A–F). Furthermore, the silencing of OIP5-AS1 using si-OIP5-AS1 did not affect HUVEC viability, apoptosis, migration, and IL-6, TNF-α, and MDA expressions (Supplementary Figures 3A–G).

### OIP5-AS1 Acted as a Sponge of miR-135a-5p

**Figure 3:**
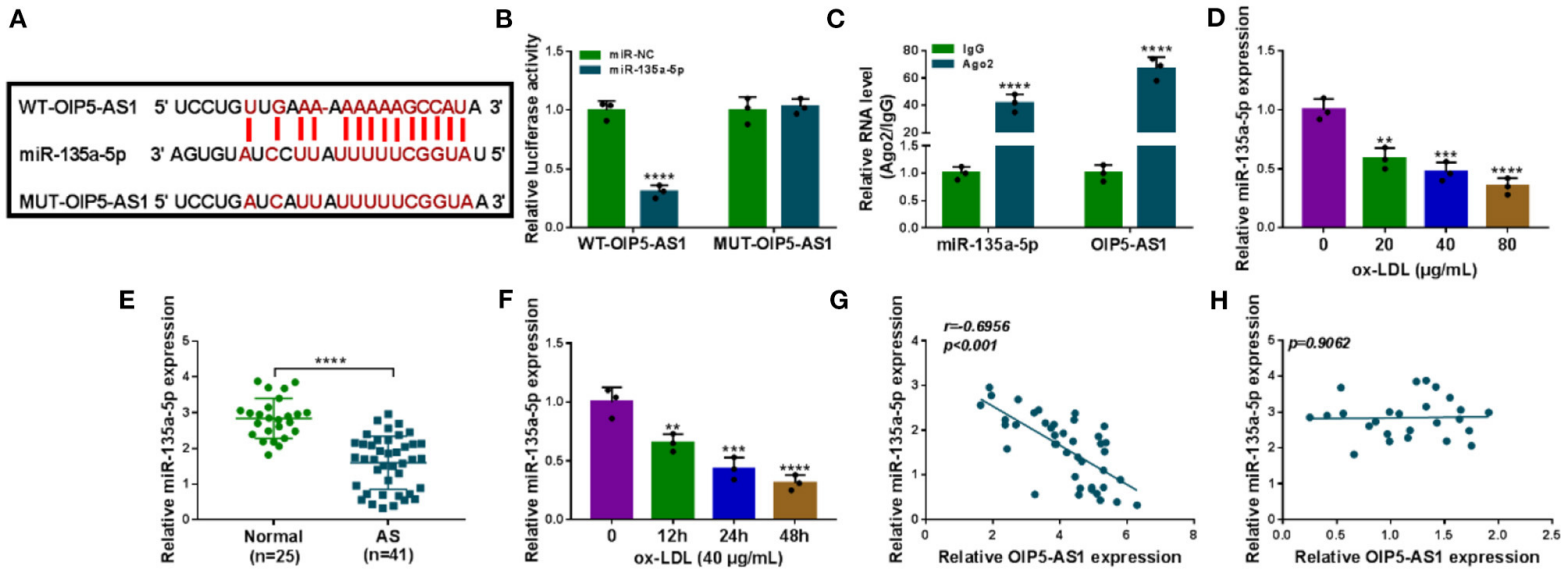
OIP5-AS1 acted as a miR-135a-5p sponge. **(A)** Schematic model of the miR-135a-5p-binding sites within OIP5-AS1 identified by starBase v.2 software and mutated miR-135a-5p-binding sites. **(B)** Relative luciferase activity in HUVECs cotransfected with WT-OIP5-AS1 or MUT-OIP5-AS1 and miR-135a-5p mimic or miR-NC mimic. *n* = 3 independent biological replicates; error bars represented SD; ****P < 0.0001 by Student's *t*-test. **(C)** The levels of miR-135a-5p and OIP5-AS1 by qRT-PCR in cell lysates incubated with anti-Ago2 or anti-IgG antibody. *n* = 3 independent biological replicates; error bars represented SD; ****P < 0.0001 by Student's *t*-test. MiR-135a-5p level by qRT-PCR in 41 atherosclerosis serum samples and 25 normal controls **(D)**, HUVECs after stimulation with 20, 40, and 80 μg/ml of ox-LDL for 24 h **(E)**, HUVECs after 40 μg/ml of ox-LDL treatment for 12, 24, and 48 h **(F)**. Error bars represented SD; **P < 0.01, ***P < 0.001, or ****P < 0.0001 by Student's *t*-test or one-way ANOVA with Tukey's *post hoc* test. Correlation between miR-135a-5p expression and OIP5-AS1 level in AS serum samples **(G)** and control serum **(H)** using the Spearman test.

To understand the mechanism by which OIP5-AS1 regulated ox-LDL-triggered cell injury, we used the starBase v.2 software to help identify the miRNAs that potentially bound to OIP5-AS1. Among these predicted candidates, miR-135a-5p was of particular interest owing to its low expression in AS and anti-atherosclerotic function in ox-LDL-stimulated HUVECs (13). The predicted data showed a putative binding sequence for miR-135a-5p within OIP5-AS1 (Figure 3A). To verify this, we performed dual-luciferase reporter assays using OIP5-AS1 luciferase reporter plasmid (WT-OIP5-AS1). WT-OIP5-AS1 and miR-135a-5p overexpression caused a downregulation in the luciferase activity (*P* < 0.0001, Figure 3B). To validate whether the miR-135a-5p-binding sites were required for this effect, an OIP5-AS1 reporter mutant (MUT-OIP5-AS1), in which all the predicted complementary sites were mutated, was tested. Notably, the mutant no longer elicited such an effect (Figure 3B). MiRNAs silence gene expression in the cytoplasm in the RNA-induced silencing complex (RISC) that also contains Ago2, a key component of the RISC (17). Using anti-Ago2 antibody, the RIP experiments showed that the enrichment levels of miR-135a-5p (*P* < 0.0001) and OIP5-AS1 (*P* < 0.0001) were synchronously elevated (Figure 3C). By contrast, miR-135a-5p was significantly underexpressed in AS serum (*P* < 0.0001) and ox-LDL-stimulated HUVECs (*P* = 0.0016, *P* = 0.0003, or *P* < 0.0001) (Figures 3D–F). Moreover, a strong inverse correlation between miR-135a-5p and OIP5-AS1 expression was observed in AS serum samples (Figure 3G). Additionally, our data showed that there was no correlation between miR-135a-5p and OIP5-AS1 expressions in control serum (Figure 3H).

### OIP5-AS1 Silencing Mitigated Ox-LDL-Triggered HUVEC Damage by Upregulating MiR-135a-5p

**Figure 4:**
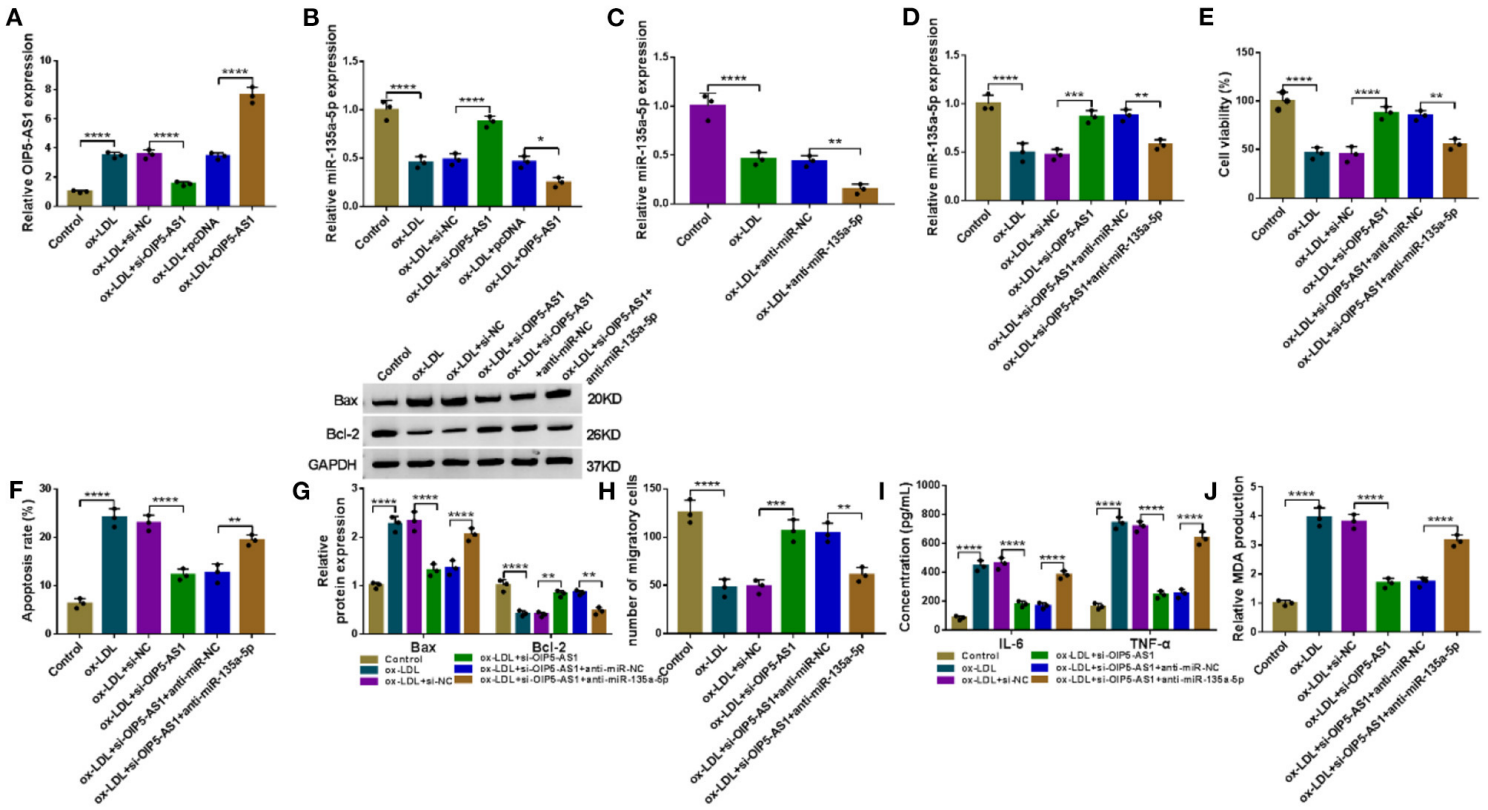
OIP5-AS1 silencing mitigated ox-LDL-triggered HUVEC damage by upregulating miR-135a-5p. The levels of OIP5-AS1 **(A)** and miR-135a-5p **(B)** by qRT-PCR in HUVECs transfected with si-NC, si-OIP5-AS1, pcDNA, or OIP5-AS1 before ox-LDL stimulation. pcDNA: negative control, OIP5-AS1: OIP5-AS1-overexpressing plasmid. **(C)** MiR-135a-5p expression by qRT-PCR in HUVECs transfected with anti-miR-NC or anti-miR-135a-5p before ox-LDL stimulation. MiR-135a-5p expression by qRT-PCR **(D)**, cell viability by CCK-8 assay **(E)**, cell apoptosis by flow cytometry **(F)**, Bax and Bcl-2 level by western blot **(G)**, cell migration by Transwell assay **(H)**, and IL-6, TNF-α, and MDA levels by ELISA **(I,J)** in HUVECs transfected with si-NC, si-OIP5-AS1, si-OIP5-AS1+anti-miR-NC, or si-OIP5-AS1+anti-miR-135a-5p before ox-LDL stimulation (40 μg/ml, 24 h). *n* = 3 independent biological replicates; error bars represented SD; *P < 0.05, **P < 0.01, ***P < 0.001, or ****P < 0.0001 by one-way ANOVA with Tukey's *post hoc* test.

We also asked whether OIP5-AS1 influenced miR-135a-5p expression in ox-LDL-stimulated HUVECs. The transfection efficiencies of si-OIP5-AS1 (*P* < 0.0001) and OIP5-AS1 overexpressing plasmid (*P* < 0.0001) were gauged by qRT-PCR in ox-LDL-treated HUVECs (Figure 4A). By contrast, miR-135a-5p level was increased by OIP5-AS1 silencing (*P* < 0.0001) and decreased by OIP5-AS1 overexpression (*P* = 0.0209) in ox-LDL-stimulated HUVECs (Figure 4B). Additionally, our data showed that OIP5-AS1 expression in HUVECs was not affected by the alteration of miR-135a-5p expression (Supplementary Figure 4).

To further determine whether miR-135a-5p was a molecular mediator of OIP5-AS1 function, we reduced miR-135a-5p expression using anti-miR-135a-5p in OIP5-AS1-silencing HUVECs under ox-LDL stimulation (*P* = 0.0023, Figures 4C,D). Functional analyses revealed that the reduced expression of miR-135a-5p abolished si-OIP5-AS1-mediated cell viability promotion (*P* = 0.0019, Figure 4E), apoptosis suppression (*P* < 0.01 or *P* < 0.0001, Figures 4F,G) and migration enhancement (*P* = 0.0037, Figure 4H) in ox-LDL-stimulated HUVECs. Furthermore, the reduced expression of miR-135a-5p dramatically abolished the reduced impact of OIP5-AS1 silencing on IL-6 and TNF-α (*P* < 0.0001, Figure 4I) and MDA (*P* < 0.0001, Figure 4J) levels in ox-LDL-stimulated HUVECs.

### MiR-135a-5p in HUVECs Directly Interacted With the 3′UTR of KLF5

**Figure 5:**
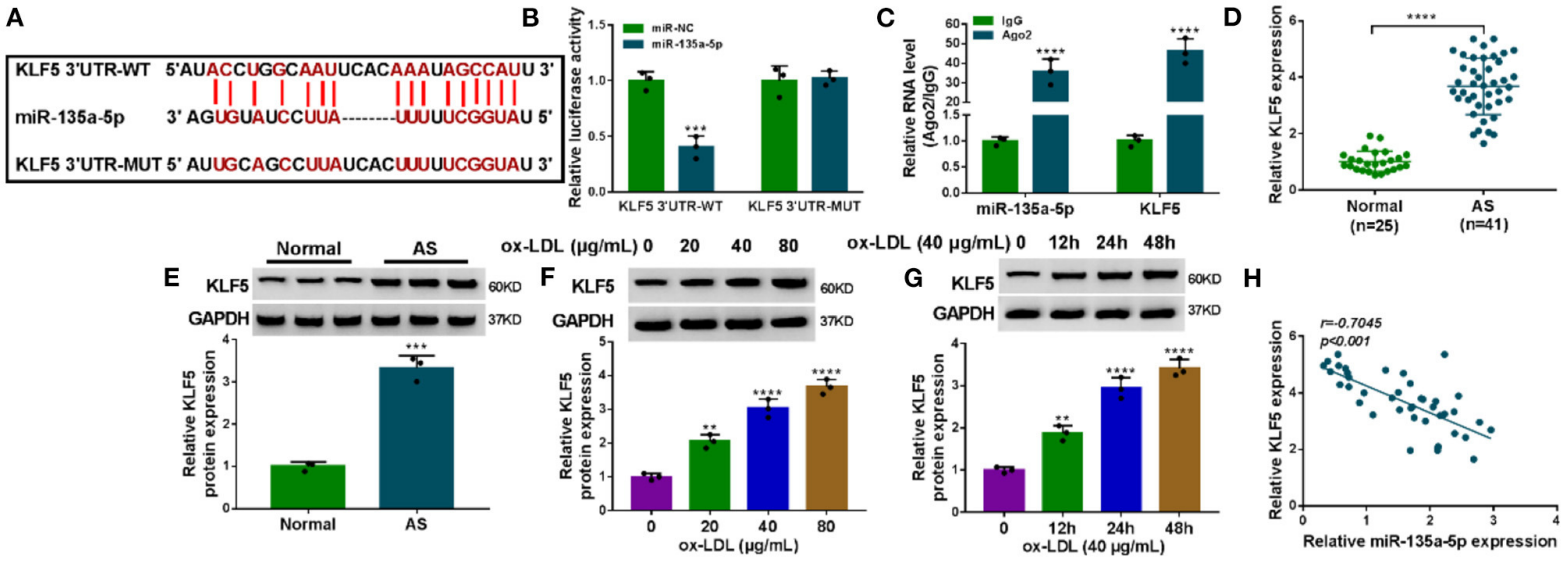
KLF5 was a direct target of miR-135a-5p in HUVECs. **(A)** Schematic of the target sequence for miR-135a-5p within KLF5 3′UTR and mutated miR-135a-5p-binding sites. **(B)** Relative luciferase activity in HUVECs cotransfected with KLF5 3′UTR-WT or KLF5 3′UTR-MUT and miR-NC mimic or miR-135a-5p mimic. *n* = 3 independent biological replicates; error bars represented SD; ***P < 0.001 by Student's *t*-test. **(C)** The enrichments of miR-135a-5p and KLF5 by qRT-PCR in cell lysates incubated with anti-Ago2 or anti-IgG antibody. *n* = 3 independent biological replicates; error bars represented SD; ****P < 0.0001 by Student's *t*-test. KLF5 level by qRT-PCR or western blot in atherosclerosis serum samples and normal controls **(D,E)**, HUVECs after stimulation with 20, 40, and 80 μg/ml of ox-LDL for 24 h **(F)**, HUVECs after 40 μg/ml of ox-LDL treatment for 12, 24, and 48 h **(G)**. Error bars represented SD; **P < 0.01, ***P < 0.001, or ****P < 0.0001 by Student's *t*-test or one-way ANOVA with Tukey's *post hoc* test. **(H)** Correlation between KLF5 expression and miR-135a-5p level in AS serum samples using the Spearman test.

To identify downstream effectors of miR-135a-5p, we used the starBase v.2 software. Among these candidates, we selected four genes (CLIC4, TXNIP, PAPPA, and KLF5) that were associated with AS pathogenesis. qRT-PCR data showed that KLF5 was the most significantly downregulated in miR-135a-5p-overexpressing cells (Supplementary Figure 5). We thus selected KLF5 for further analyses. To validate whether KLF5 was a direct target of miR-135a-5p, we used KLF5 3′UTR luciferase reporter (KLF5 3′UTR-WT) and its mutation (KLF5 3′UTR-MUT) (Figure 5A) in luciferase assays. The KLF5 3′UTR-WT and miR-135a-5p mimic caused a downregulation in luciferase activity (*P* = 0.0003, Figure 5B). When the target sequence was mutated, little reduction in luciferase was observed in the presence of miR-135a-5p mimic (Figure 5B). RIP analyses showed that the enrichment levels of miR-135a-5p and KLF5 were simultaneously elevated by anti-Ago2 antibody (*P* < 0.0001, Figure 5C). Additionally, KLF5 was overexpressed in AS serum and ox-LDL-stimulated HUVECs (Figures 5D–G). Of interest, in AS serum samples, an inverse correlation between KLF5 expression and miR-135a-5p level was discovered (Figure 5H).

### MiR-135a-5p Overexpression Relieved Ox-LDL-Triggered HUVEC Injury by Downregulating KLF5

**Figure 6:**
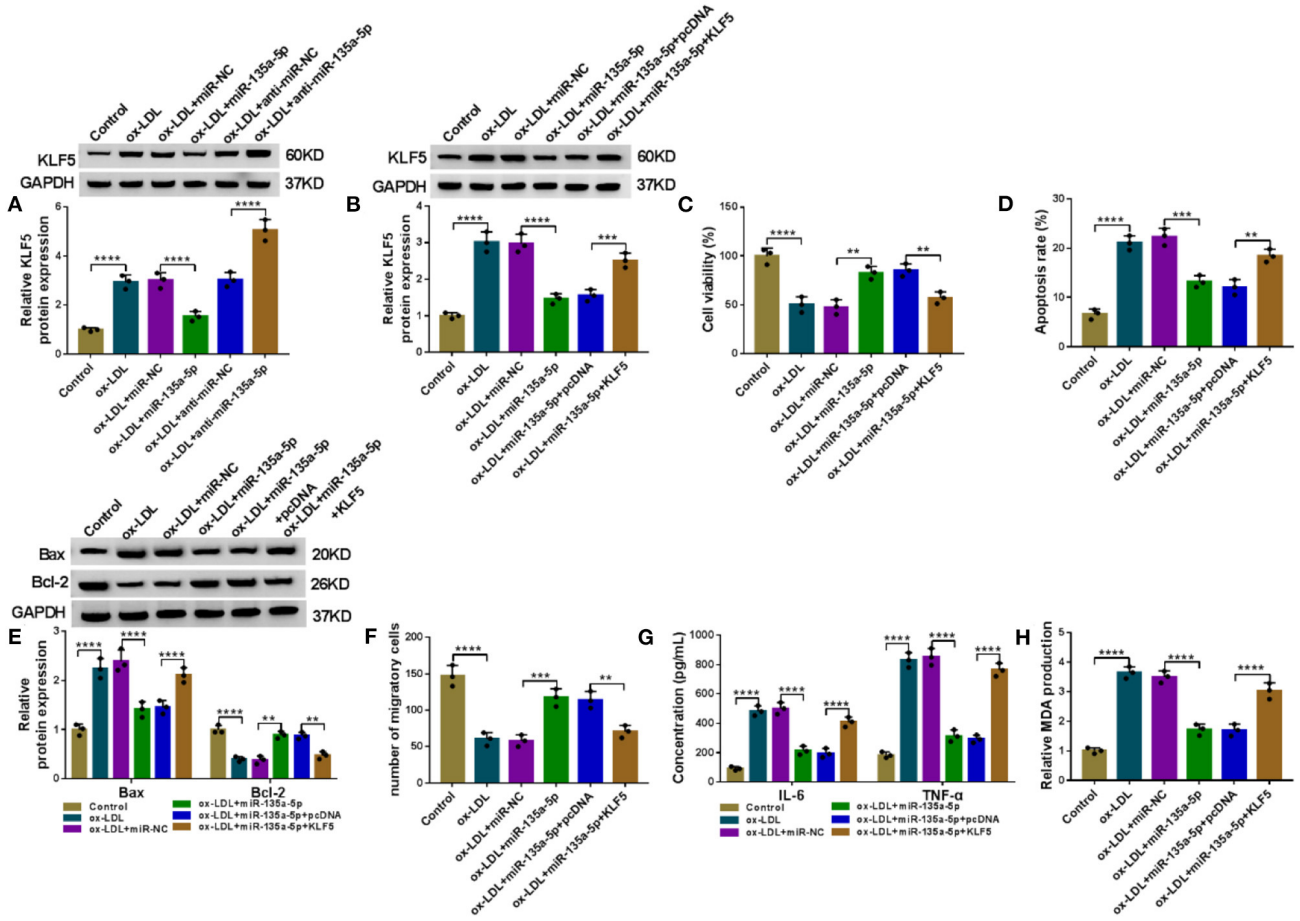
The alleviative effects of miR-135a-5p overexpression on ox-LDL-triggered HUVEC injury were abated by KLF5 expression restoration. **(A)** KLF5 protein expression determined by western blot in HUVECs transfected with miR-NC mimic, miR-135a-5p mimic, anti-miR-NC, or anti-miR-135a-5p before stimulation with ox-LDL (40 μg/ml, 24 h). HUVECs were transfected with miR-NC mimic, miR-135a-5p mimic, miR-135a-5p mimic+pcDNA, or miR-135a-5p mimic+KLF5 before ox-LDL stimulation (40 μg/ml, 24 h), followed by the determination of KLF5 protein level by western blot **(B)**, cell viability by CCK-8 assay **(C)**, cell apoptosis by flow cytometry **(D)**, Bax and Bcl-2 levels by western blot **(E)**, cell migration by Transwell assay **(F)**, and IL-6, TNF-α, and MDA levels by ELISA **(G,H)**. pcDNA: negative control, KLF5: KLF5-overexpressing plasmid. *n* = 3 independent biological replicates; error bars represented SD; **P < 0.01, ***P < 0.001, or ****P < 0.0001 by one-way ANOVA with Tukey's *post hoc* test.

A crucial question was whether miR-135a-5p modulated KLF5 expression in HUVECs. As expected, in contrast to the corresponding negative control, the KLF5 protein level was reduced by miR-135a-5p overexpression (*P* < 0.0001), and it was elevated by anti-miR-135a-5p in HUVECs under ox-LDL stimulation (*P* < 0.0001, Figure 6A). To examine whether KLF5 was a functional target of miR-135a-5p in regulating ox-LDL-induced injury, we overexpressed KLF5 using a KLF5-overexpressing plasmid in ox-LDL-treated HUVECs transfected with miR-135a-5p mimic (*P* = 0.0009, Figure 6B). Functional experiments showed that the enforced expression of miR-135a-5p led to a promotion in cell viability (*P* = 0.0016, Figure 6C), a repression in cell apoptosis (Figures 6D,E), and an enhancement in cell migration (*P* = 0.001, Figure 6F), as well as a strong reduction in IL-6 and TNF-α (*P* < 0.0001, Figure 6G) and MDA (*P* < 0.0001, Figure 6H) levels in HUVECs under ox-LDL stimulation. Nevertheless, these effects of miR-135a-5p overexpression were abrogated by KLF5 expression restoration in ox-LDL-stimulated HUVECs (*P* < 0.01 or *P* < 0.0001, Figures 6C–H).

### OIP5-AS1 Regulated KLF5 Expression by Acting as a MiR-135a-5p Sponge

**Figure 7:**
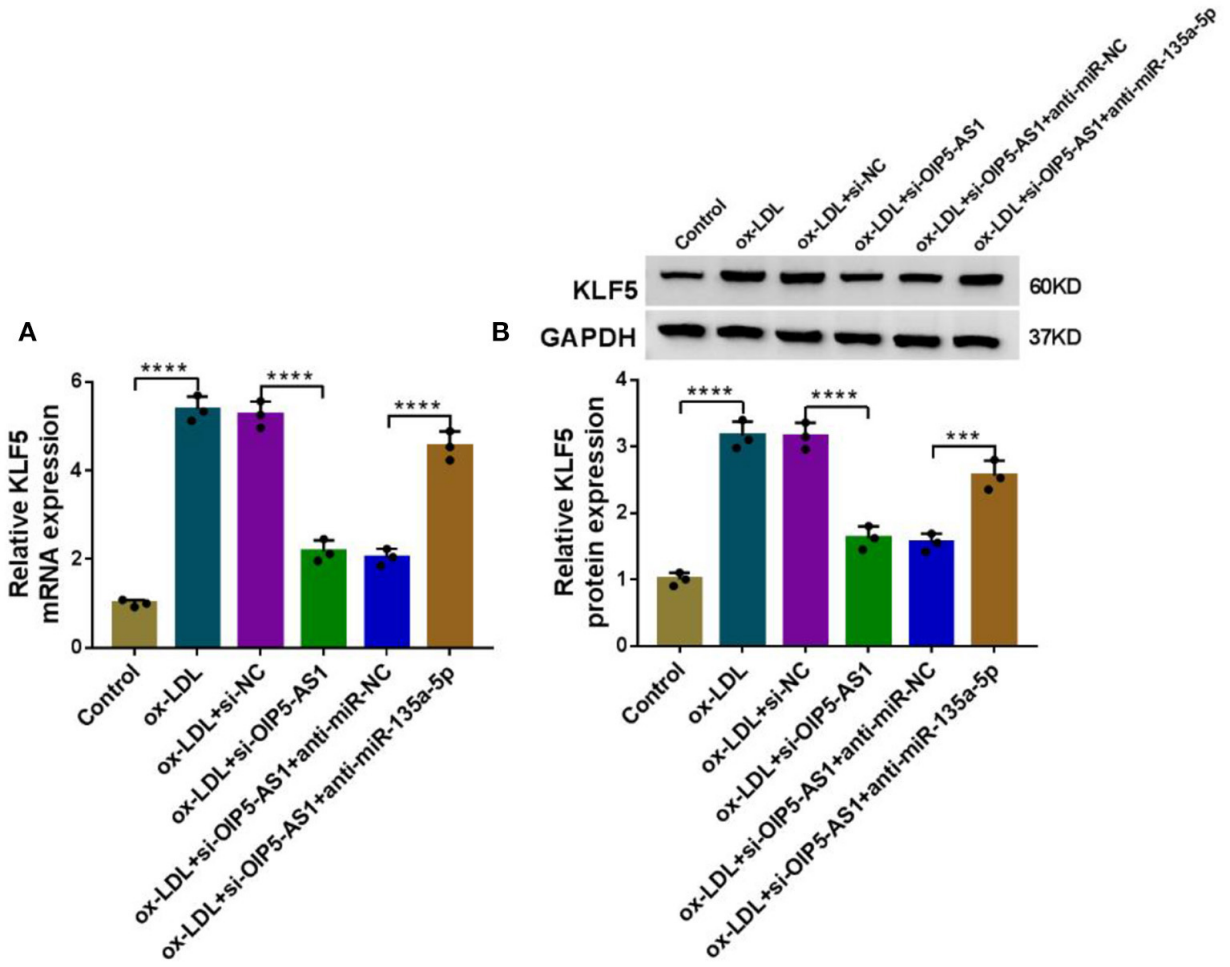
OIP5-AS1 regulated KLF5 expression through sponging miR-135a-5p. KLF5 mRNA expression determined by qRT-PCR **(A)** and KLF5 protein level by western blot **(B)** in HUVECs transfected with si-NC, si-OIP5-AS1, si-OIP5-AS1+anti-miR-NC, or si-OIP5-AS1+anti-miR-135a-5p before stimulation with ox-LDL (40 μg/ml, 24 h). *n* = 3 independent biological replicates; error bars represented SD; ***P < 0.001 or ****P < 0.0001 by one-way ANOVA with Tukey's *post hoc* test.

We next ascertained whether OIP5-AS1 influenced KLF5 expression through functioning as a miRNA sponge. By contrast, KLF5 mRNA and protein levels were reduced by OIP5-AS1 silencing (*P* < 0.0001), while these effects were dramatically abolished by anti-miR-135a-5p in ox-LDL-stimulated HUVECs (*P* < 0.001 or *P* < 0.0001, Figures 7A,B).

### Silencing of OIP5-AS1 Diminished the Levels of Total Cholesterol, Triglyceride, HDL-C, and LDL-C in ApoE^–/–^ Mice

**Figure 8:**
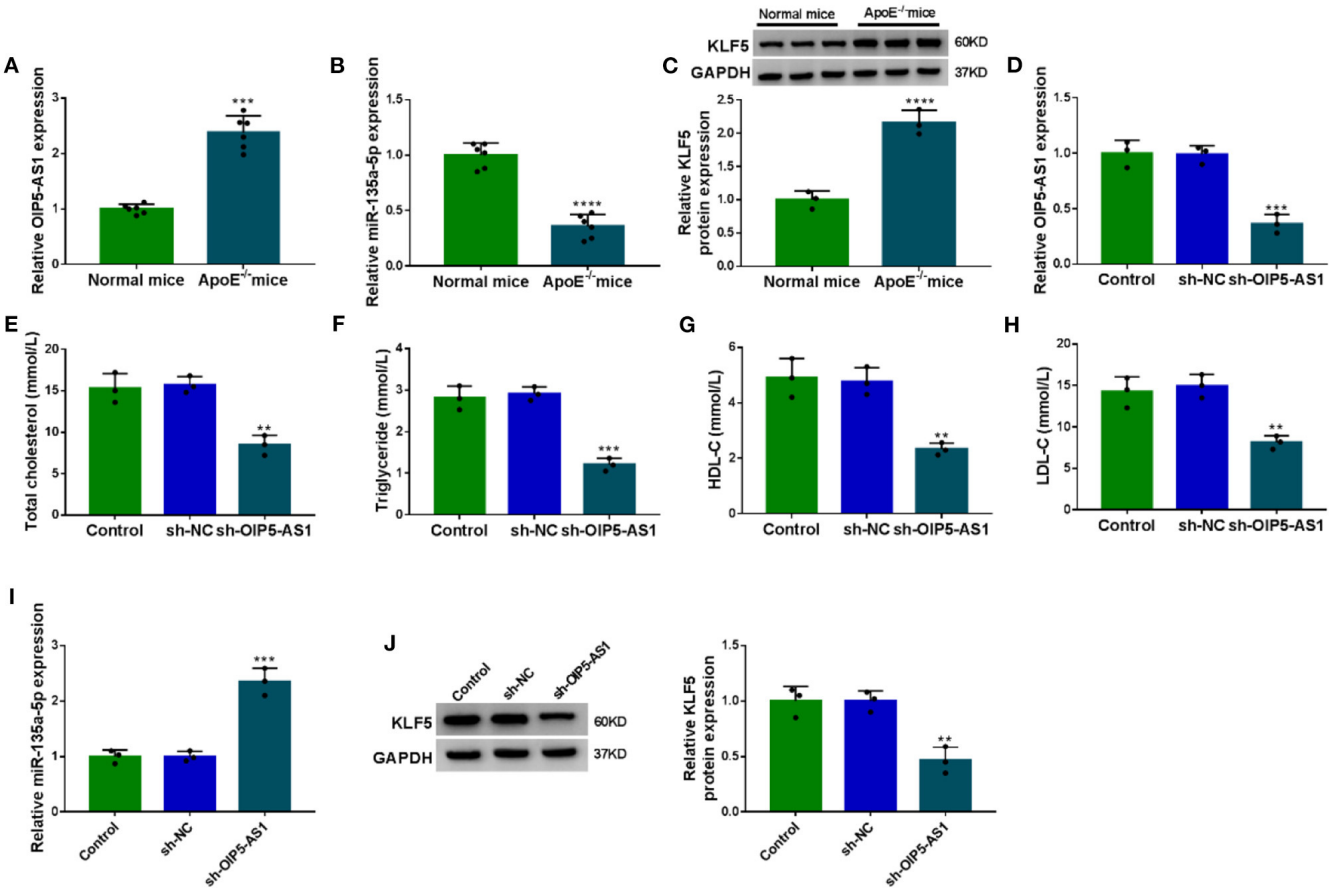
The silencing of OIP5-AS1 weakened atherosclerosis progression in ApoE^−/−^ mice. ApoE^−/−^ mice were fed with a high-fat diet and wild-type C57BL/6J mice (normal mice) were fed with normal chow. In the 6th week, the ApoE^−/−^ mice were injected with PBS (control), sh-NC, or sh-OIP5-AS1 by caudal vein every week until the 12th week (*n* = 6 per group). At the end of the experiments, serum samples were harvested from all mice. **(A–D)** OIP5-AS1, miR-135a-5p, and KLF5 expression levels determined by qRT-PCR and western blot in the serum samples. **(E–H)** The levels of total cholesterol, triglyceride, HDL-C, and LDL-C in the serum samples from ApoE^−/−^ mice. **(I,J)** The levels of miR-135a-5p and KLF5 by qRT-PCR and western blot in the serum samples. *n* = 6 independent biological replicates; error bars represented SD; **P < 0.01, ***P < 0.001, or ****P < 0.0001 by Student's *t*-test or one-way ANOVA with Tukey's *post hoc* test.

Lastly, we evaluated whether OIP5-AS1 could influence atherosclerosis progression *in vivo*. Interestingly, by contrast, OIP5-AS1 level was upregulated in the serum samples of ApoE^−/−^ mice fed with high-fat diet (*P* = 0.0007, Figure 8A). Moreover, miR-135a-5p expression was downregulated (*P* < 0.0001, Figure 8B), and KLF5 level was upregulated (*P* = 0.0009, Figure 8C) in serum of ApoE^−/−^ mice fed with high-fat diet. Remarkably, the infection with sh-OIP5-AS1 led to a decrease in the level of OIP5-AS1 in the serum samples (*P* = 0.0005, Figure 8D). More importantly, the reduced expression of OIP5-AS1 dramatically diminished the levels of total cholesterol, triglyceride, HDL-C, and LDL-C in the serum samples of ApoE^−/−^ mice fed with high-fat diet (*P* < 0.001 or *P* < 0.0001, Figures 8E–H). Additionally, the reduced expression of OIP5-AS1 led to an increase in miR-135a-5p expression (*P* = 0.0001, Figure 8I) and a decrease in KLF5 level (*P* = 0.0032, Figure 8J) in the serum samples.

## Discussion

Emerging evidence has shown a diversity of lncRNA-mediated mechanisms that are involved in atherosclerosis pathogenesis, particularly highlighting the lncRNA/miRNA/mRNA network (18, 19). Here, we first identified the OIP5-AS1/miR-135a-5p/KLF5 regulatory network in ox-LDL-mediated pro-atherogenic effect in HUVECs.

In this report, our data showed the overexpression of OIP5-AS1 in atherosclerosis serum and ox-LDL-stimulated HUVECs, in line with previous studies (9, 10). High expression of OIP5-AS1 has been found in various cancers, such as thyroid cancer, gastric cancer, and oral squamous cell carcinoma (20–22). Moreover, the increased level of OIP5-AS1 influenced hemangioma vascular endothelial cell proliferation by regulating NIN1/RPN12 binding protein 1 homolog (NOB1) expression by acting as a miR-195-5p sponge (23). OIP5-AS1 was also identified to be associated with the pathogenesis of multiple sclerosis (24). In this report, we showed that OIP5-AS1 knockdown protected HUVECs from ox-LDL-triggered cytotoxicity and diminished atherosclerosis progression in ApoE^−/−^ mice, consistent with previous studies (9, 10).

Here, we first demonstrated that OIP5-AS1 acted as a miR-135a-5p sponge. MiR-135a-5p has been identified as a key modulator in a series of human cancers, including glioblastoma, HCV-associated hepatocellular carcinoma, and gallbladder cancer (25–27). Moreover, miR-135a-5p has been implicated to osteoporosis progression, cerebral hypoxia/reoxygenation-evoked damage, and epilepsy development (28–30). Our results showed that the enforced expression of miR-135a-5p ameliorated HUVEC damage induced by ox-LDL, in agreement with a previous study (13). Moreover, we first demonstrated the alleviative effect of OIP5-AS1 silencing on ox-LDL-induced HUVEC injury through miR-135a-5p. The findings by Zhang et al. illuminated that OIP5-AS1 served as a sponge of miR-320a to modulate ox-LDL-induced HUVEC injury by influencing LOX1 expression (10).

Subsequently, we first identified KLF5 as a direct target of miR-135a-5p in HUVECs. KLF5, a zinc-finger transcription factor, is highly expressed in AS and is associated with the malignant symptomatology of atherosclerosis (31–33). In this report, we first showed the anti-atherosclerotic effect of miR-135a-5p overexpression in ox-LDL-induced HUVECs through KLF5. Similarly, Wang and colleagues reported that miR-152 protected against atherosclerosis malignant progression by directly targeting KLF5 (34). Our data also pointed out the role of OIP5-AS1 as a miR-135a-5p sponge to modulate KLF5 in HUVECs under ox-LDL. Similarly, smooth muscle-enriched lncRNA (SMILR) enhanced atherosclerosis development by modulating KLF5 expression by sponging miR-10b-3p (35). Zheng et al. reported that vascular smooth muscle cell-derived exosomes could mediate the transfer of KLF5-induced miR-155 from smooth muscle cells to endothelial cells, and the overexpression of miR-155 suppressed endothelial cell proliferation and migration, leading to atherosclerosis progression (36). Wang et al. demonstrated that KLF5 overexpression induced by hyperinsulinemia contributed to diabetic endothelial dysfunction by regulating endothelial nitric oxide synthase (eNOS) (37). Future work should build on these findings by determining precisely the molecular determinants of KLF5 in regulating ox-LDL-induced injury in HUVECs. Additionally, HAECs are atherosclerosis-related aortic endothelial cells that are widely used to establish the atherosclerosis *in vitro* model (38, 39). Our data also showed that ox-LDL induced a strong increase in OIP5-AS1 expression in HAECs, and the reduced expression of OIP5-AS1 abolished ox-LDL-induced injury in HAECs (Supplementary Figures 1A–F), reinforcing OIP5-AS1 as a novel therapeutic target for atherosclerosis.

In conclusion, we identified a novel regulatory network, the OIP5-AS1/miR-135a-5p/KLF5 axis, in ox-LDL-induced HUVEC dysfunction. We provided evidence that OIP5-AS1, a significantly overexpressed lncRNA in atherosclerosis, regulated ox-LDL-triggered HUVEC damage by targeting the miR-135a-5p/KLF5 axis, highlighting a promising molecular target for atherosclerosis management.

## Data Availability Statement

The original contributions presented in the study are included in the article/Supplementary Material, further inquiries can be directed to the corresponding author/s.

## Author Contributions

MZ conducted the experiments and drafted the manuscript. YY designed the study and edited the manuscript. JL conducted the experiments and supervised the study. ML collected and analyzed the data. YW contributed the methodology. All authors read and approved the final manuscript.

## Conflict of Interest

The authors declare that the research was conducted in the absence of any commercial or financial relationships that could be construed as a potential conflict of interest.

## Supplementary Material

Supplementary Figure 1 The effect of OIP5-AS1 on ox-LDL-triggered injury in HAECs. **(A)** Relative OIP5-AS1 expression in HAECs treated with 20, 40, and 80 μg/ml of ox-LDL for 24 h. HAECs were transfected with si-NC or si-OIP5-AS1 before ox-LDL treatment, followed by the determination of OIP5-AS1 level by qRT-PCR **(B)**, cell viability by CCK-8 assay **(C)**, cell apoptosis by flow cytometry **(D)**, and IL-6, TNF-α, and MDA levels by ELISA **(E,F)**. *n* = 3 independent biological replicates; error bars represented SD; ^**^*P* < 0.01, ^***^*P* < 0.001, or ^****^*P* < 0.0001 by one-way ANOVA with Tukey's *post hoc* test.

Supplementary Figure 2 The enforced expression of OIP5-AS1 regulated cell viability, apoptosis, and expression of IL-6, TNF-α, and MDA in HUVECs. HUVECs were transfected with or without pcDNA or OIP5-AS1-overexpressing plasmid, followed by the assessment of OIP5-AS1 expression by qRT-PCR **(A)**, cell viability by CCK-8 assay **(B)**, cell apoptosis by flow cytometry **(C)**, Bax and Bcl-2 levels by western blot **(D)**, and IL-6, TNF-α, and MDA levels by ELISA **(E,F)**. *n* = 3 independent biological replicates; error bars represented SD; ^**^*P* < 0.01, ^***^*P* < 0.001 or ^****^*P* < 0.0001 by one-way ANOVA with Tukey's *post hoc* test.

Supplementary Figure 3 Effect of OIP5-AS1 knockdown on cell viability, apoptosis, migration, and expression of IL-6, TNF-α, and MDA in HUVECs. HUVECs were treated with control or ox-LDL or transfected with si-NC or si-OIP5-AS1, followed by the assessment of OIP5-AS1 expression by qRT-PCR **(A)**, cell viability by CCK-8 assay **(B)**, cell apoptosis by flow cytometry **(C)**, cell migration by Transwell assay **(D)**, Bax and Bcl-2 level by western blot **(E)**, and IL-6, TNF-α, and MDA levels by ELISA **(F,G)**. *n* = 3 independent biological replicates; error bars represented SD; ^***^*P* < 0.001, ^****^*P* < 0.0001 or NS: not significant by one-way ANOVA with Tukey's *post hoc* test.

Supplementary Figure 4 The impact of miR-135a-5p level on OIP5-AS1 expression. HUVECs were transfected with miR-NC mimic, miR-135a-5p mimic, anti-miR-NC, or anti-miR-135a-5p, followed by the detection of OIP5-AS1 expression by qRT-PCR. *n* = 3 independent biological replicates; error bars represented SD; *P* > 0.05 by one-way ANOVA with Tukey's *post hoc* test.

Supplementary Figure 5 The expression of CLIC4, TXNIP, PAPPA, and KLF5 by qRT-PCR in HUVECs transfected with miR-NC mimic or miR-135a-5p mimic. *n* = 3 independent biological replicates; error bars represented SD; ^**^*P* < 0.01 or ^****^*P* < 0.0001 by Student's *t*-test.
