# Supplementary figures and images for: Silencing of OIP5-AS1 Protects Endothelial Cells From ox-LDL-Triggered Injury by Regulating KLF5 Expression via Sponging miR-135a-5p
Source: Front Cardiovasc Med. 2021 Mar 12;8:596506. doi: 10.3389/fcvm.2021.596506 (PMC7994260; doi:10.3389/fcvm.2021.596506)

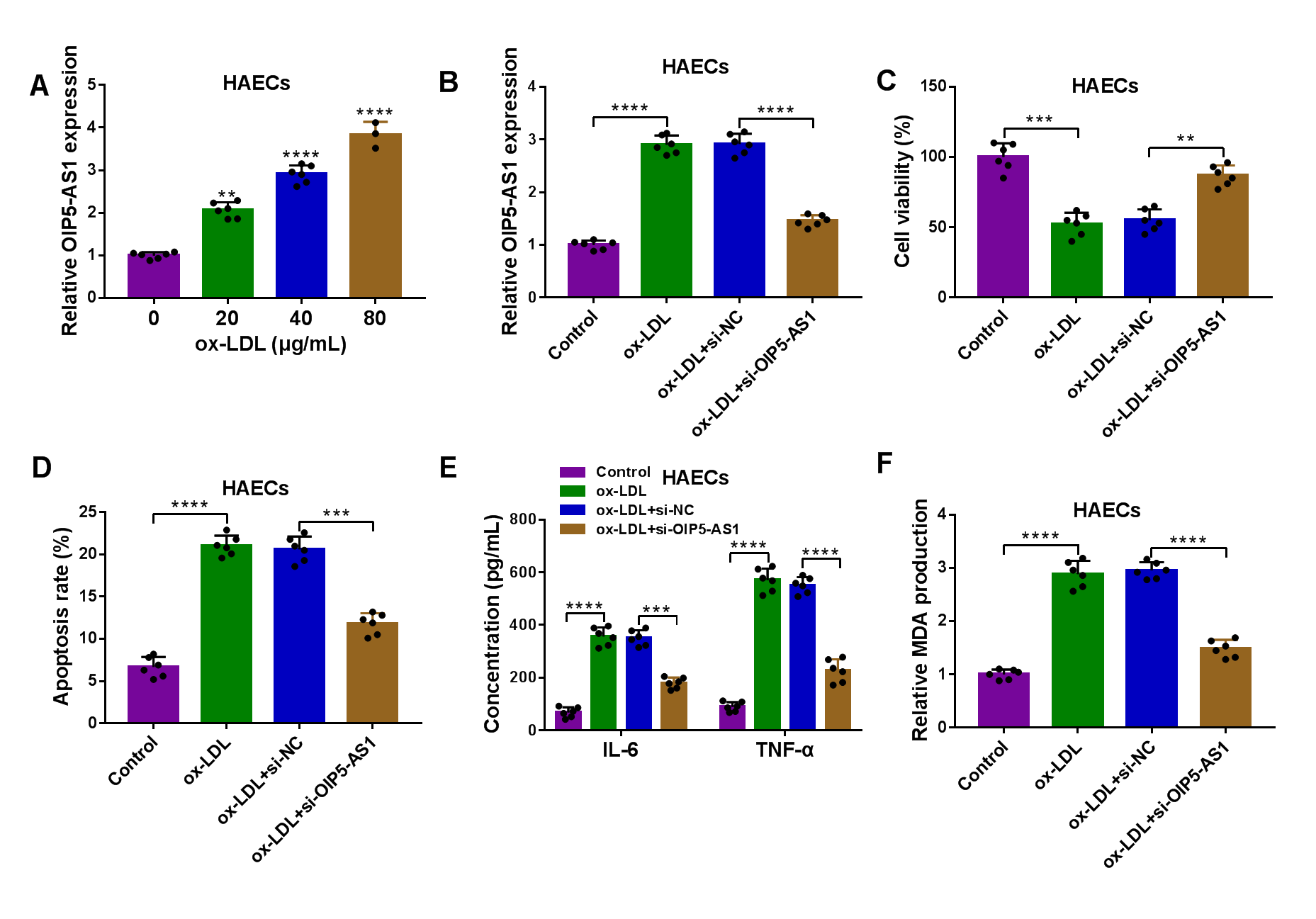

Supplement: Supplementary Figure 1 — The effect of OIP5-AS1 on ox-LDL-triggered injury in HAECs. (A) Relative OIP5-AS1 expression in HAECs treated with 20, 40, and 80 μg/ml of ox-LDL for 24 h. HAECs were transfected with si-NC or si-OIP5-AS1 before ox-LDL treatment, followed by the determination of OIP5-AS1 level by qRT-PCR (B), cell viability by CCK-8 assay (C), cell apoptosis by flow cytometry (D), and IL-6, TNF-α, and MDA levels by ELISA (E,F). n = 3 independent biological replicates; error bars represented SD; **P < 0.01, ***P < 0.001, or ****P < 0.0001 by one-way ANOVA with Tukey's post hoc test. [file Image_1.TIF]

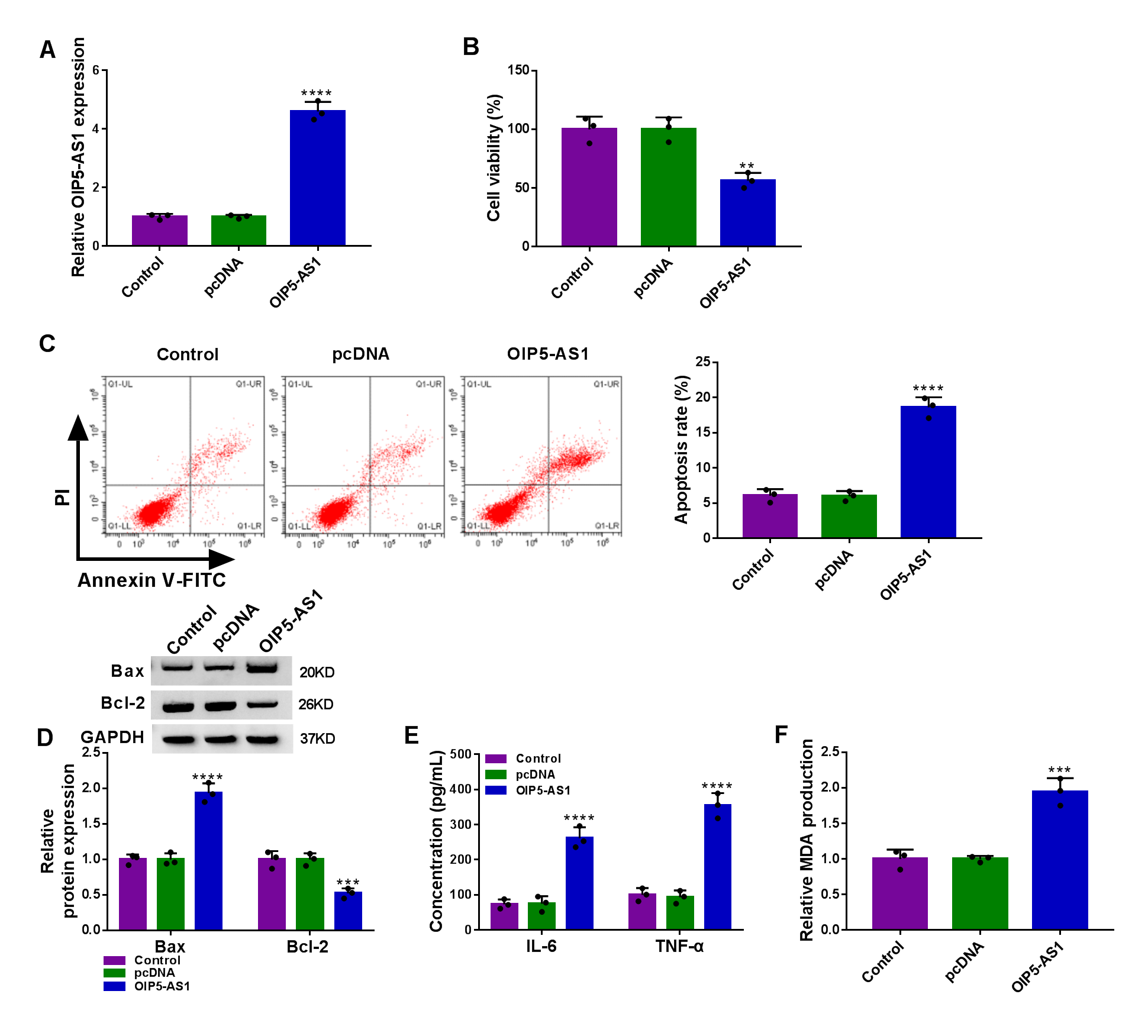

Supplement: Supplementary Figure 2 — The enforced expression of OIP5-AS1 regulated cell viability, apoptosis, and expression of IL-6, TNF-α, and MDA in HUVECs. HUVECs were transfected with or without pcDNA or OIP5-AS1-overexpressing plasmid, followed by the assessment of OIP5-AS1 expression by qRT-PCR (A), cell viability by CCK-8 assay (B), cell apoptosis by flow cytometry (C), Bax and Bcl-2 levels by western blot (D), and IL-6, TNF-α, and MDA levels by ELISA (E,F). n = 3 independent biological replicates; error bars represented SD; **P < 0.01, ***P < 0.001 or ****P < 0.0001 by one-way ANOVA with Tukey's post hoc test. [file Image_2.TIF]

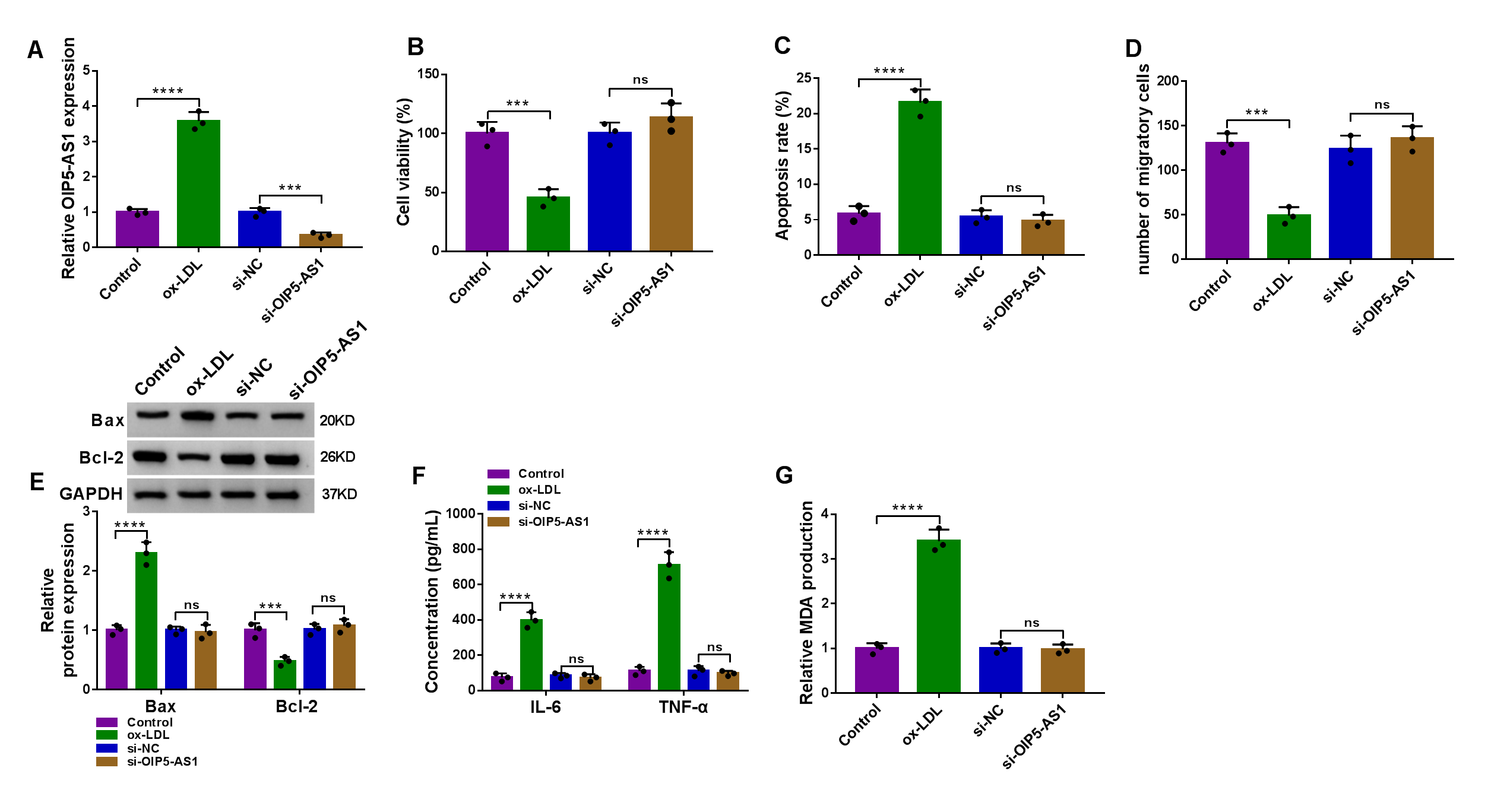

Supplement: Supplementary Figure 3 — Effect of OIP5-AS1 knockdown on cell viability, apoptosis, migration, and expression of IL-6, TNF-α, and MDA in HUVECs. HUVECs were treated with control or ox-LDL or transfected with si-NC or si-OIP5-AS1, followed by the assessment of OIP5-AS1 expression by qRT-PCR (A), cell viability by CCK-8 assay (B), cell apoptosis by flow cytometry (C), cell migration by Transwell assay (D), Bax and Bcl-2 level by western blot (E), and IL-6, TNF-α, and MDA levels by ELISA (F,G). n = 3 independent biological replicates; error bars represented SD; ***P < 0.001, ****P < 0.0001 or NS: not significant by one-way ANOVA with Tukey's post hoc test. [file Image_3.TIF]

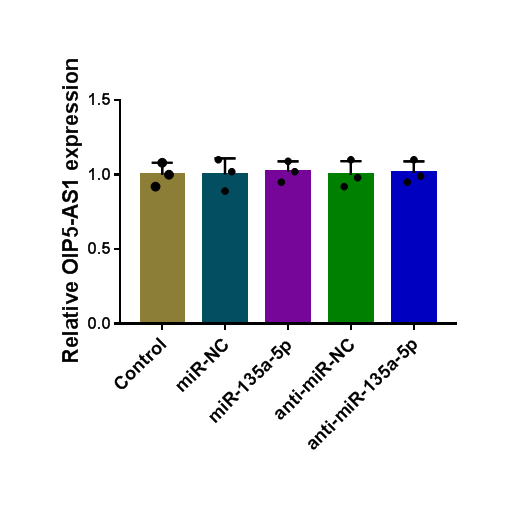

Supplement: Supplementary Figure 4 — The impact of miR-135a-5p level on OIP5-AS1 expression. HUVECs were transfected with miR-NC mimic, miR-135a-5p mimic, anti-miR-NC, or anti-miR-135a-5p, followed by the detection of OIP5-AS1 expression by qRT-PCR. n = 3 independent biological replicates; error bars represented SD; P > 0.05 by one-way ANOVA with Tukey's post hoc test. [file Image_4.TIF]

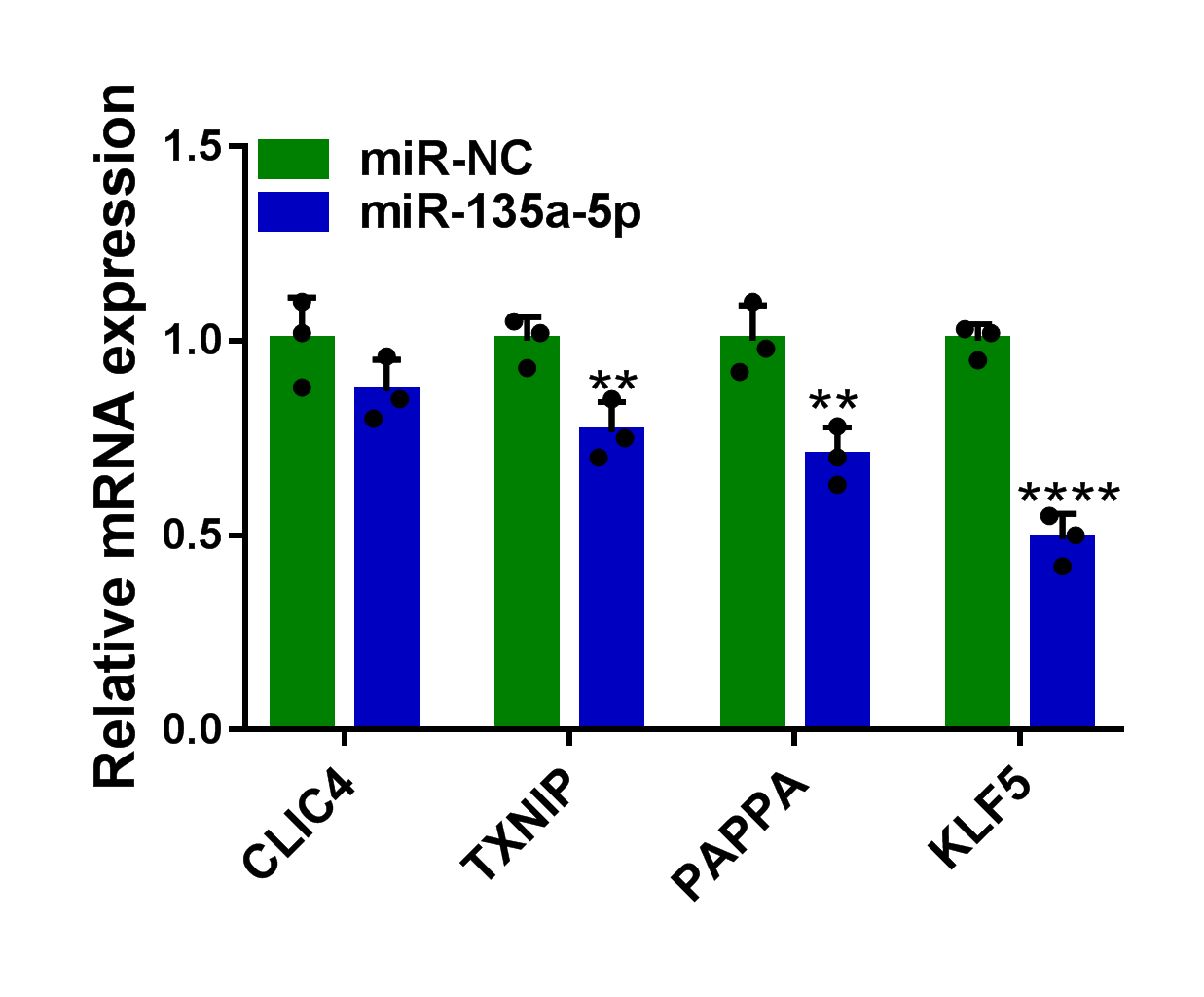

Supplement: Supplementary Figure 5 — The expression of CLIC4, TXNIP, PAPPA, and KLF5 by qRT-PCR in HUVECs transfected with miR-NC mimic or miR-135a-5p mimic. n = 3 independent biological replicates; error bars represented SD; **P < 0.01 or ****P < 0.0001 by Student's t-test. [file Image_5.TIF]
